# *MC4R* Variants Modulate α-MSH and Setmelanotide Induced Cellular Signaling at Multiple Levels

**DOI:** 10.1210/clinem/dgae210

**Published:** 2024-04-03

**Authors:** Alejandra V Rodríguez Rondón, Mila S Welling, Erica L T van den Akker, Elisabeth F C van Rossum, Elles M J Boon, Mieke M van Haelst, Patric J D Delhanty, Jenny A Visser

**Affiliations:** Obesity Center CGG and Expertise Center Genetic Obesity, Erasmus MC, University Medical Center Rotterdam, 3015 GD Rotterdam, The Netherlands; Department of Internal Medicine, Division of Endocrinology, Erasmus MC, University Medical Center Rotterdam, 3015 GD Rotterdam, The Netherlands; Obesity Center CGG and Expertise Center Genetic Obesity, Erasmus MC, University Medical Center Rotterdam, 3015 GD Rotterdam, The Netherlands; Department of Internal Medicine, Division of Endocrinology, Erasmus MC, University Medical Center Rotterdam, 3015 GD Rotterdam, The Netherlands; Department of Pediatrics, Division of Endocrinology, Erasmus MC-Sophia Children's Hospital, University Medical Center Rotterdam, 3015 GD Rotterdam, The Netherlands; Obesity Center CGG and Expertise Center Genetic Obesity, Erasmus MC, University Medical Center Rotterdam, 3015 GD Rotterdam, The Netherlands; Department of Pediatrics, Division of Endocrinology, Erasmus MC-Sophia Children's Hospital, University Medical Center Rotterdam, 3015 GD Rotterdam, The Netherlands; Obesity Center CGG and Expertise Center Genetic Obesity, Erasmus MC, University Medical Center Rotterdam, 3015 GD Rotterdam, The Netherlands; Department of Internal Medicine, Division of Endocrinology, Erasmus MC, University Medical Center Rotterdam, 3015 GD Rotterdam, The Netherlands; Department of Human Genetics, Amsterdam University Medical Center, 1105 AZ Amsterdam, The Netherlands; Department of Human Genetics, Amsterdam University Medical Center, 1105 AZ Amsterdam, The Netherlands; Obesity Center CGG and Expertise Center Genetic Obesity, Erasmus MC, University Medical Center Rotterdam, 3015 GD Rotterdam, The Netherlands; Department of Internal Medicine, Division of Endocrinology, Erasmus MC, University Medical Center Rotterdam, 3015 GD Rotterdam, The Netherlands; Obesity Center CGG and Expertise Center Genetic Obesity, Erasmus MC, University Medical Center Rotterdam, 3015 GD Rotterdam, The Netherlands; Department of Internal Medicine, Division of Endocrinology, Erasmus MC, University Medical Center Rotterdam, 3015 GD Rotterdam, The Netherlands

**Keywords:** melanocortin-4 receptor, genetic variation, obesity, setmelanotide, α-MSH, G-protein-coupled receptors, β-arrestins

## Abstract

**Context:**

The melanocortin-4 receptor (*MC4R*) plays an important role in body weight regulation. Pathogenic *MC4R* variants are the most common cause of monogenic obesity.

**Objective:**

We have identified 17 *MC4R* variants in adult and pediatric patients with obesity. Here we aimed to functionally characterize these variants by analyzing 4 different aspects of MC4R signaling. In addition, we aimed to analyze the effect of setmelanotide, a potent MC4R agonist, on these MC4R variants.

**Materials and Methods:**

Cell surface expression and α-melanocyte stimulating hormone (α-MSH)- or setmelanotide-induced cAMP response, β-arrestin-2 recruitment, and ERK activation were measured in cells expressing either wild type or variant MC4R.

**Results:**

We found a large heterogeneity in the function of these variants. We identified variants with a loss of response for all studied MC4R signaling, variants with no cAMP accumulation or ERK activation but normal β-arrestin-2 recruitment, and variants with normal cAMP accumulation and ERK activation but decreased β-arrestin-2 recruitment, indicating disrupted desensitization and signaling mechanisms. Setmelanotide displayed a greater potency and similar efficacy as α-MSH and induced significantly increased maximal cAMP responses of several variants compared to α-MSH. Despite the heterogeneity in functional response, there was no apparent difference in the obesity phenotype in our patients.

**Conclusion:**

We show that these obesity-associated *MC4R* variants affect MC4R signaling differently yet lead to a comparable clinical phenotype. Our results demonstrate the clinical importance of assessing the effect of *MC4R* variants on a range of molecular signaling mechanisms to determine their association with obesity, which may aid in improving personalized treatment.

Obesity is a public health concern that is caused by an imbalance in energy homeostasis and is associated with multiple comorbidities, including type 2 diabetes, cardiovascular disease, various types of cancer, infertility, and depression (1-3). The obesogenic environment and a sedentary lifestyle contribute largely to obesity. However, there is also a genetic component to the regulation of body weight. Based on multiple twin studies, the hereditability of body weight is estimated at 40% to 70% (4, 5). The predisposition to obesity is mainly polygenic, but in a significant minority of patients with obesity it can be monogenic (2, 6). Monogenic obesity results mainly from pathogenic variants in genes affecting the evolutionarily conserved leptin-melanocortin pathway, the appetite regulatory system in the hypothalamus (7, 8). One of these genes is the melanocortin-4 receptor (*MC4R*), and pathogenic variants in *MC4R* are the most frequent cause of monogenic obesity (9, 10).

Loss of function (LoF) *MC4R* variants are the most common monogenic cause of obesity. Affected individuals present with early-onset obesity, hyperphagia, hyperinsulinemia, increased bone mineral density, and increased linear growth (4, 11, 12). The prevalence of LoF *MC4R* variants ranges from 0.5% in adults with obesity to 5.8% in children with severe early-onset obesity (13, 14). Moreover, the prevalence of heterozygous *MC4R* carriers in Europe with a body mass index (BMI) greater than 30 kg/m^2^ is 1% to 2.5% (14, 15). Also in mice, homozygous deficiency of *Mc4r* results in severe obesity and hyperphagia, with heterozygous mice having an intermediate phenotype (16). These findings indicate that MC4R is a major regulator of appetite and energy balance in the leptin-melanocortin pathway (17, 18).

*MC4R* is expressed in neurons of the paraventricular nucleus of the hypothalamus and signals predominantly through the Gα_s_ protein/cAMP pathway in response to the ligand α-melanocyte stimulating hormone (α-MSH) (9, 10). Its activation leads to a reduction in food intake and an increase in energy expenditure (9, 10, 19, 20). α-MSH is produced locally by pro-opiomelanocortin (POMC) neurons, through processing of the POMC polypeptide precursor, in response to leptin (9, 10, 19, 20).

More than 200 *MC4R* variants have been identified and associated with obesity (21). The majority of the identified *MC4R* variants follow an autosomal dominant (monoallelic) inheritance pattern, and the pathogenic variant receptors show a LoF in Gα_s_ signaling (22, 23). Most of these variants cause misfolding of the protein, leading to retention and/or degradation of the receptor by the quality control system (endoplasmic reticulum or Golgi apparatus), preventing receptor trafficking to the cell surface (24, 25). However, some obesity-associated MC4R variants signal normally via cAMP, which has raised the possibility that they perturb other uncharacterized signaling pathways linking MC4R to the regulation of food intake (26).

In addition to the Gα_s_/cAMP pathway, MC4R has been shown to signal through β-arrestins (27). Recruitment of β-arrestin to the activated receptor leads to desensitization and internalization of the receptor into the cytosol but also may modulate the activation of the ERK pathway (28-30). Indeed, knocking down *ARRB2*, which encodes β-arrestin-2, increased ERK1/2 phosphorylation in a cell model. Moreover, knocking down *GNAS*, which encodes the Gα_s_ protein, resulted in a complete inhibition of ERK1/2 phosphorylation (30). This data, among other studies, suggests that MC4R-induced ERK signaling, possibly through Gαs, may also contribute to the regulation of food intake (30-33).

Given the important role of MC4R in energy metabolism, MC4R is considered a potential drug target for the treatment of genetic obesity (34-36). Setmelanotide (also named RM-493, BIM-22493, IRC-022493, and brand name Imcivree) is a synthetic cyclic MC4R agonist approved by the Food and Drug Administration and European Medicines Agency and shown to be effective in reducing body weight in patients with obesity caused by bi-allelic pathogenic *POMC, PCSK1*, or *LEPR* variants (35, 36), as well as in diet-induced animal models (35). Setmelanotide has a high affinity and specificity for MC4R (35-37). Interestingly, setmelanotide also induced weight loss in *Mc4r* heterozygous knockout mice upon high-fat diet, while homozygous knockout mice were nonresponsive. Although the effect of setmelanotide in the *Mc4r* heterozygous knockout mice was less compared to wild type (WT) mice (35), it may suggest that patients with a pathogenic *MC4R* variant might benefit from treatment with setmelanotide. This may be particularly relevant since carriers of heterozygous LoF *MC4R* variants respond poorly to diet- and exercise-induced weight loss (35). Although only a few studies have been performed, results suggest that indeed setmelanotide may potentiate weight loss in patients with heterozygous *MC4R* variants, albeit that patients with variants upstream of the MC4R pathway (eg, POMC) are more responsive (35).

Here we have functionally analyzed 17 *MC4R* variants (1 homozygous, 1 compound heterozygous, and 15 heterozygous), identified in patients with severe obesity attending our Obesity Center CGG at Erasmus MC, Rotterdam, the Netherlands. We sought to gain more insight into the impact of these obesity-associated variants on various aspects of MC4R signaling: cell surface expression; total receptor expression; and α-MSH-induced cAMP production, β-arrestin-2 recruitment, and ERK pathway activation. In addition, we studied whether these variants were responsive to setmelanotide and compared functional results with aspects of their clinical phenotype.

## Materials and Methods

### Clinical Data

#### Patient cohort and sequencing analysis

Patient sample collection and sequencing analysis were performed by the Genome Diagnostics section of the Department of Genetics, UMC Utrecht, the Netherlands, and by the Department of Human Genetics, Amsterdam UMC, the Netherlands (2). DNA samples were analyzed through diagnostic next-generation sequencing for genetic obesity disorders (obesitome). Additional DNA diagnostics, including whole exome sequencing, single-nucleotide polymorphism array, and karyotyping, were performed due to additional clinical features, such as developmental delay, intellectual deficit, short stature, or autism [Supplementary Table S1 (38)]. The study involving human participants was reviewed and approved by the Ethical Committee of the Erasmus MC, University Medical Centre, Rotterdam (pediatric study MEC-2012-257 and adult study MEC-2023-0029). Informed consent of all participants and/or caregivers was obtained, when needed, according to the approved protocol.

The *MC4R* variants were identified using obesity gene panels whose content evolved during the course of this study (2, 39). *MC4R* variants were classified based on assessment of the available evidence, in line with the guidelines of the American College of Medical Genetics and Genomics (ACMG) Laboratory Practice Committee Working Group (40), integrated with our professional expertise and knowledge of the genes and disorders we routinely offer diagnostic service for. Variants were classified into 1 of the 5 following categories: pathogenic, likely pathogenic, unknown/uncertain significance, likely benign or benign.

#### Resting energy expenditure measurement

Resting energy expenditure (REE) was measured after an overnight fast using indirect calorimetry with a metabolic cart (Quark RMR, COSMED, Italy, for children and Q-NRG, COSMED, Italy, for adults). The Schofield equations were used to calculate predicted REE in children <18 years (41, 42). The predicted REE for patients ≥18 years was calculated using the 1984 Harris–Benedict equations (43, 44).

#### Body composition measurement

In children, body composition was measured using air displacement plethysmography (BOD POD, COSMED, Italy). The Lohman density model was used to measure the two-compartment body composition (fat-free mass and fat mass) from body volume, according to the manufacturer's recommendation (45). For adults, body composition was measured using bio-impedance analysis (InBody S10, the Netherlands).

### Functional Analysis

#### Cell lines, transfection, cloning, and site-directed mutagenesis

For all experiments, human embryonic kidney 293 (HEK293) cells (ECACC cat. no. 85120602, RRID: CVCL_0045, UK) were cultured in DMEM/F12 (GIBCO, USA) containing 2.5 mM GlutaMAX, 10% fetal calf serum (FCS), 100 units/mL penicillin, and 100 µg/mL streptomycin (P/S) (all media reagents from Thermo Fisher Scientific, the Netherlands) at 37 °C in humidified air containing 5% CO_2_.

The pcDNA3.1(+)-MC4R expression plasmid, containing the WT human *MC4R* cDNA was obtained from the cDNA Resource Center (www.cdna.org, USA). For the GloSensor cAMP assay, pcDNA3.1(+) expression plasmid containing WT or variant MC4R and the GloSensor 22F plasmid (Promega, USA) were used to measure cAMP accumulation. For the β-arrestin-2 recruitment assay, the cDNA of human ARRB2 (β-arrestin-2; SinoBiological Europe GmbH, Germany) was cloned into the pBiT2.1-N [TK/SmBiT] vector, generating an N-terminally tagged ARRB2, and the cDNA of human MC4R was cloned into pBiT1.1-C [TK/LgBiT] vector (Promega, USA), generating a C-terminally LgBiT-tagged MC4R. For cell surface expression, the *MC4R* cDNA was cloned into pBiT3.1-secN [CMV/HiBiT/Blast] adding an IL-6 secretory signal peptide and HiBiT to its N-terminus (Promega, USA). The variants were generated in all of these constructs by site-directed mutagenesis, using the QuikChange II XL kit (Agilent Technologies, USA) according to the manufacturer's protocols [primers are listed in Supplementary Table S2 (38)]. Sanger sequencing was used to verify all constructs.

Transfections were performed using FuGENE HD (Promega, USA) or Lipofectamine 3000 (Thermo Fisher, the Netherlands) in serum-free Opti-MEM medium (Thermo Fisher, the Netherlands) according to the manufacturer's protocol.

#### Measurement of cAMP levels

HEK293 cells (1.3 × 10^6^) were seeded into 25 cm^2^ culture flasks (Greiner Bio-One, Austria) and transiently transfected with 0.2 µg of WT or variant *MC4R* expression plasmid and 2 µg of pGloSensor-22F cAMP plasmid. The next day, cells were reseeded into a clear-bottom 96-well plate (100 µL/well) (Greiner Bio-One). After 24 hours, culture media was replaced by 50 µL of 2% GloSensor cAMP reagent (Promega, USA) in CO_2_-independent serum-free stimulation medium (GIBCO, USA) containing P/S and 0.1% BSA and incubated for 2 hours at 37 °C. Levels of cAMP were measured using the GloSensor cAMP assay (Promega, USA) according to the manufacturer's protocols. Baseline luminescence was measured for 10 minutes using the Victor X4 plate reader. Next, cells were stimulated with α-MSH (Tocris, UK) or setmelanotide (MedChem Express, the Netherlands) at concentrations ranging from 10^−11^ to 10^−6^ M for 20 minutes and luminescence was measured.

Independent experiments were performed to compare the cAMP response of α-MSH and setmelanotide on WT MC4R, according to the protocol described previously, except that luminescence was measured using the CLARIOstar Plus reader (BMG LABTECH, Germany). The assessment of the WT MC4R cAMP response was performed separately from that of the MC4R variants, explaining the variation in median effective concentration (EC_50_).

#### Measurement of β-arrestin-2 recruitment

The interaction between MC4R and β-arrestin-2 was measured using the Nano-Glo Live Cell Assay System (Promega, USA). Briefly, 40 000 cells/well were seeded in poly-D-lysine-coated (Sigma Aldrich, USA) clear-bottom white 96-well plates, transiently transfected with 50 ng/well of each of the constructs using Lipofectamine 3000, and incubated overnight at 37 °C in culture medium. The next day, the culture medium was replaced with a CO_2_-independent medium (with 0.5% FCS and P/S), and Nano-Glo Live Cell Substrate (Promega, USA) was added to the wells and cells were temperature equilibrated for 10 minutes inside the Victor X4 plate reader (Perkin Elmer, USA) before baseline measurement. Next, cells were stimulated with α-MSH or setmelanotide at concentrations ranging from 10^−11^ to 10^−5^ M. Cells were incubated for 4 minutes before the luminescent signal was measured.

Separate experiments were performed to directly compare α-MSH and setmelanotide induced β-arrestin-2 recruitment by WT MC4R, according to the protocol described previously, except that luminescence was measured using the CLARIOstar Plus reader (BMG LABTECH, Germany). This may explain the difference in EC_50_ from experiments in which the variants were evaluated.

#### Quantification of extracellular and total expression

Cell surface expression and total expression of WT and variant MC4R were measured using the Nano-Glo HiBiT Extracellular Detection System and Nano-Glo HiBiT Lytic Detection System respectively (Promega, USA). In brief, 20 000 cells/well were seeded in poly-D-lysine-coated clear-bottom white 96-well plates. The next day, cells were transiently transfected with 0.25 ng/well of the constructs. After 24 hours of transfection, the culture media was substituted for a CO_2_ independent medium (0.5% FCS and P/S), and 50 µL of the HiBiT Extracellular or Lytic reagent was added into the wells. Cells were incubated for 10 minutes at room temperature and then luminescence was measured.

#### Measurement of ERK pathway activation

To assess the impact of MC4R variants on ERK activation, a serum response element (SRE) luciferase reporter plasmid was used (pGL4.33[luc2/SRE/Hygro]; Promega, USA). HEK293 cells were seeded on poly-D-lysine-coated white clear-bottom 96-well plates (20 000 cells/well) and cotransfected with the SRE reporter plasmid (50 ng/well), WT, or variant *MC4R* expression plasmid (50 ng/well) and the pSV40-RL plasmid (10 ng/well) (Promega, USA). After 24 hours, cells were starved overnight in 50 µL/well CO_2_-independent medium (0.1% BSA and P/S), then stimulated with α-MSH or setmelanotide at concentrations ranging from 10^−11^ to 10^−5^ M for 5 hours at 37 °C and 5% CO_2_. The stimulation was terminated by removing the media from the wells and lysing the cells with 25 µL/well lysis buffer. Plates were placed on a shaker for 10 minutes at room temperature before storage at −20 °C. Luciferase activity was measured using the Dual-Glo Luciferase Assay System (Promega, USA) in the CLARIOstar Plus reader (BMG LABTECH, Germany).

Separate experiments were performed to compare α-MSH and setmelanotide induced ERK activation through WT MC4R, according to the protocol described previously. This may explain the difference in EC_50_ from experiments in which the variants were evaluated.

#### Statistical analysis

GraphPad Prism (version 9.0.0 for Windows; RRID: SCR_002798; CA, USA) was used to perform nonlinear curve fitting of the dose-response data, to calculate EC_50_, and for all statistical tests. Comparison between WT and variant MC4R were assessed statistically using unpaired two-tailed *t*-tests, and a *P*-value <.05 was considered significant. Results are derived from 3 to 4 independent experiments using duplicate samples. Data for MC4R variants are expressed relative to WT MC4R (set at 100%).

The variants were categorized into 4 groups based on their mean %Emax cAMP response to α-MSH relative to WT: complete LoF (cLoF, <20% of WT), partial LoF (pLoF, 20-80% of WT), WT-like (80-120% of WT), and gain of function (GoF, >120% of WT).

## Results

### Clinical Phenotype of Patients With Variants in MC4R

**Figure 1.**
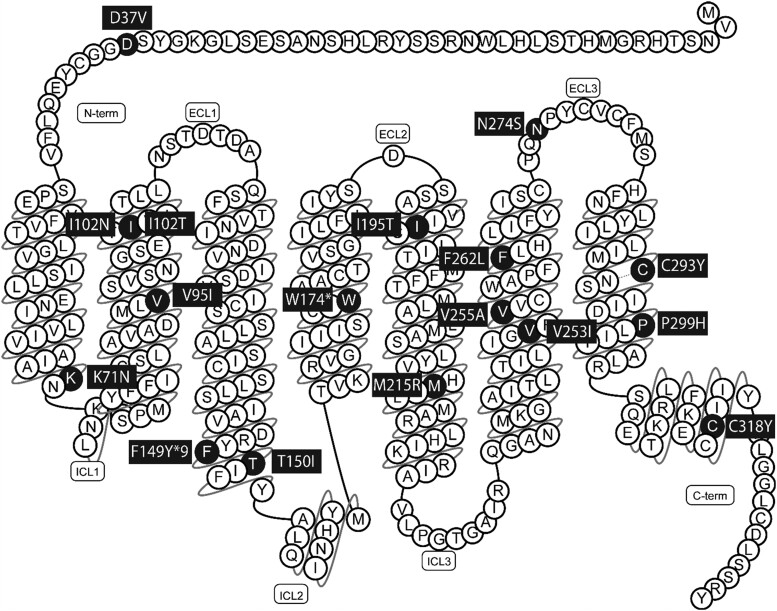
Schematic model of MC4R. The identified variants (n = 17) in patients with obesity are highlighted in black and labeled with the respective amino acid substitution, frameshift, or deletion. (Snake diagram from https://gpcrdb.org/protein/mc4r_human/). Abbreviations: ECL, extracellular loop; ICL, intracellular loop; MC4R, melanocortin-4 receptor.

One homozygous, 1 compound heterozygous, and 15 heterozygous *MC4R* variants were identified upon screening using an obesity gene panel in unrelated adult and pediatric patients of the outpatient clinic Obesity Center CGG, Erasmus MC: D37V, **K71N**, V95I, **I102N**, I102T, F149YfsX9, T150I, W174X, I195T, M215R, V253I, V255A, **F262L**, N274S, **C293Y**, P299H, and **C318Y** (Fig. 1 and Table 1). Variants in bold are novel variants. Underlined variants have been identified previously but were not functionally characterized. The other variants were assessed in part previously for cAMP response, ligand binding, and/or cell surface expression. Nucleotide changes of these *MC4R* variants are listed in Table 1. All variants were identified and assessed in silico for pathogenicity according to the ACMG guidelines (40) and reported as variants of unknown significance, likely pathogenic, or pathogenic (Table 2).

**Table 1.** Clinical phenotype of patients with MC4R variants

Amino acid change	Nucleotide change	Sex	Variant carried by parent		Zygosity	AoO obesity (years)	Height (cm)	Height (SDS)	Weight (kg)	Weight (SDS)	BMI (kg/m^2^)	BMI (SDS)	Hyperphagia		REE, kcal/day (% of reference)*^a^*	Fat mass (%)*^b,c^*
			Fa	Mo									Impaired satiety	Impaired satiation		
**Pediatric patients (n = 11)**																
K71N	213G > T	F	n.d.	n.d.	Homozygous	3.0	147.5	0.9	55.5	3.3	25.5	3.5	No	No	102.0	50.3
I102T	305T > C	M	n.d.	n.d.	Heterozygous	0.5	136.6	1.4	52.5	4.8	28.1	5.1	No	No	95.0	39.0
T150I	449C > T	M	n.d.	n.d.	Heterozygous	0.3	106.0	1.2	34.2	6.9	30.4	7.2	Yes	No	n.d.	n.d.
W174X	521G > A	M	n.d.	n.d.	Heterozygous	4.0	114.8	−0.6	29.7	2.9	22.5	4.3	Yes	Yes	111.3	38.6
I195T	584T > C	F	No	n.d.	Heterozygous	4.0	144.2	−1.8	67.7	2.4	32.6	3.5	No	No	99.8	33.9
M215R	644T > G	M	Yes	No	Heterozygous	3.0	55.5	3.3	147.5	0.9	25.5	3.5	No	No	98.8	35.5
V255A	764T > C	F	Yes	n.d.	Heterozygous	5.0	139.4	0.8	56.6	3.6	29.1	3.8	Yes	Yes	93.3	47.6
F262L	784T > C	M	n.d.	Yes	Heterozygous	3.0	174.5	−0.7	121.6	5.0	39.9	4.1	Yes	No	85.0	42.3
N274S	821A > G	M	Yes	n.d.	Heterozygous	2.8	174.6	1.7	117.0	4.5	38.4	4.2	Yes	Yes	n.d.	n.d.
C293Y	878G > A	M	Yes	n.d.	Heterozygous	1.0	176.3	2.4	85.3	3.6	27.4	3.1	Yes	Yes	117.0	n.d.
P299H	896C > A	M	n.d.	n.d.	Heterozygous	3.3	127.6	0.8	54.0	5.7	33.2	6.6	Yes	Yes	89.0	48.4
**Adult patients (n = 6)**																
D37V	110A > T	F	Yes	No	Compound heterozygous*^d^*	1.0	168.5	n.d.	167.0	n.d.	58.8	n.d.	Yes	Yes	104.2	57.8
V95I	283G > T	F	n.d.	n.d.	Heterozygous	6.0	144.2	n.d.	74.9	n.d.	36.0	n.d.	No	Yes	88.0	n.d.
I102N	305T > A	F	n.d.	n.d.	Heterozygous	6.0	180.0	n.d.	182.2	n.d.	56.2	n.d.	Yes	Yes	n.d.	57.9
F149YfsX9	446-450del	F	n.d.	n.d.	Heterozygous	4.0	166.1	n.d.	153.4	n.d.	55.6	n.d.	Yes	No	82.0	55.3
V253I	757G > A	M	n.d.	n.d.	Heterozygous	0.0	191.7	n.d.	141.7	n.d.	38.6	n.d.	Yes	No	94.6	41.0
C318Y	953G > A	F	No	Yes	Heterozygous	0.3	164.1	n.d.	113.3	n.d.	42.1	n.d.	Yes	Yes	118.0	48.5

Abbreviations: AoO, age of onset; BMI, body mass index; F, female; Fa, father; M, male; MC4R, melanocortin-4 receptor; Mo, mother; n.d, not determined; REE, resting energy expenditure.

^*a*^Measured by indirect calorimetry, reference values were calculated using the Schofield formula for children or Harris–Benedict formula for adults.

^*b*^Measured with bioelectrical impedance analysis.

^*c*^Measured with BODPOD.

^*d*^Patient also carries the pathogenic variant Y35X.

**Table 2.** MC4R variants: in silico predicted pathogenicity and functional outcome

Amino acid change	Nucleotide change	Predicted*^a^*	α-MSH induced cAMP response	Classification*^b^*
D37V*^c^*	110A > T	P	pLoF	III/IV
K71N	213G > T	US	pLoF	I/II
V95I	283G > T	P	pLoF	III/IV
I102T	305T > C	US	cLoF	I/II
I102N	305T > A	LP	cLoF	III/IV
F149YfsX9	446-450del	LP	cLoF	I
T150I	449C > T	LP	cLoF	II
W174X	521G > A	LP	cLoF	I
I195T	584T > C	US	WT-like	II/V
M215R	644T > G	US	cLoF	I
V253I	757G > A	US	pLoF	III/IV
V255A	764T > C	US	WT-like	V
F262L	784T > C	US	pLoF	III/IV
N274S	821A > G	US	WT-like	V
C293Y	878G > A	US	cLoF	I/II
P299H	896C > A	LP	cLoF	I
C318Y	953G > A	US	GoF	—

Abbreviations: cLoF, complete loss-of-function; GoF, gain-of-function; MC4R, melanocortin-4 receptor; n.d., not determined; pLoF, partial loss-of-function; WT-like, wildtype-like.

^*a*^Variants were classified into 5 categories: pathogenic (P), likely pathogenic (LP), unknown/uncertain significance (US), likely benign (LB), or benign (B).

^*b*^Molecular classification of the MC4R variants identified in patients according to Tao (56). class: (I) null mutations, (II) intracellularly trapped variants, (III) binding defective variants, (IV) signaling defective variants, and (V) variants with apparently normal function.

^*c*^Patient also carries the pathogenic variant Y35X.

The detected variants were scattered throughout the MC4R protein but were located mostly in the transmembrane domains (Fig. 1A). Of these variants, 10 heterozygous variants and 1 homozygous variant were found in pediatric patients with severe obesity (Table 1). The median BMI- SD score (SDS), which adjusts for age and sex of these patients, was +4.2 SDS (ranging from +3 +7 SDS), indicating severe obesity. Their median age of obesity onset was 3 years, indicating early onset (<5 years). Six heterozygous variants were identified in adult patients (Table 1). The median BMI of these patients was 48.8 kg/m^2^ (ranging from 36 to 58.8 kg/m^2^), and median age of obesity onset was 2.5 years. Of all patients, 12 (6 pediatric and 6 adult) presented with hyperphagia (Table 1), and 13 patients (9 pediatric and 4 adults) had obesity-related comorbidities [Supplementary Table S3 and S4 (38)]. Of these 13 patients, 10 patients were hyperinsulinemic and 7 were dyslipidemic. REE and fat mass are described in Table 1.

### Functional Analysis

#### Setmelanotide has similar efficacy but greater potency than α-MSH in stimulating the MC4R signaling pathways

**Figure 2.**
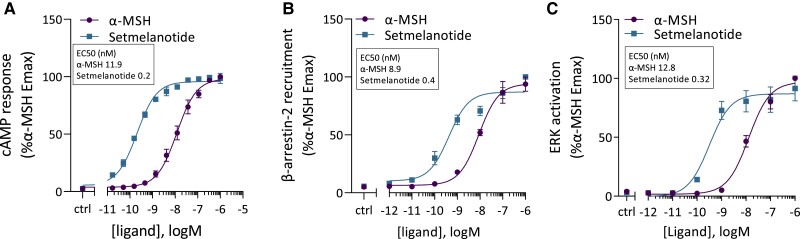
Setmelanotide has a greater potency but similar efficacy as α-MSH. Effect of α-MSH and setmelanotide on efficacy (Emax) and potency (EC_50_) of MC4R cAMP response (A), β-arrestin-2 recruitment (B), and ERK activation (C). The cAMP response is presented as Emax relative to WT MC4R upon α-MSH stimulation. Values represent mean ± SEM from 3 to 4 independent experiments performed in duplicate. Statistical analysis was performed using unpaired two-tailed *t*-test; confidence level = 95%; ^*^P ≤ .05 and **P ≤ .001. Abbreviations: α-MSH, α-melanocyte stimulating hormone; EC_50,_ median effective concentration; Emax, maximum effect; MC4R, melanocortin-4 receptor; WT, wild type.

We determined the efficacy and potency of α-MSH and setmelanotide by assessing the WT MC4R canonical Gα_s_-dependent cAMP response, β-arrestin-2 recruitment, and ERK activation (Fig. 2A–2C). The efficacy of setmelanotide in eliciting a signaling response at MC4R was similar to that of α-MSH (*P* > .05; Fig. 2A–2C). However, as reported previously (35, 46), setmelanotide had a significantly 22-fold greater potency than α-MSH for cAMP response (EC_50_: 0.2 nM vs 11.9 nM, respectively) (Fig. 2A). Setmelanotide also had a significantly 21-fold and 40-fold greater potency for β-arrestin-2 recruitment and ERK activation, respectively, compare to α-MSH (Fig. 2B and 2C).

#### MC4R variants affect ligand-induced cAMP response

**Figure 3.**
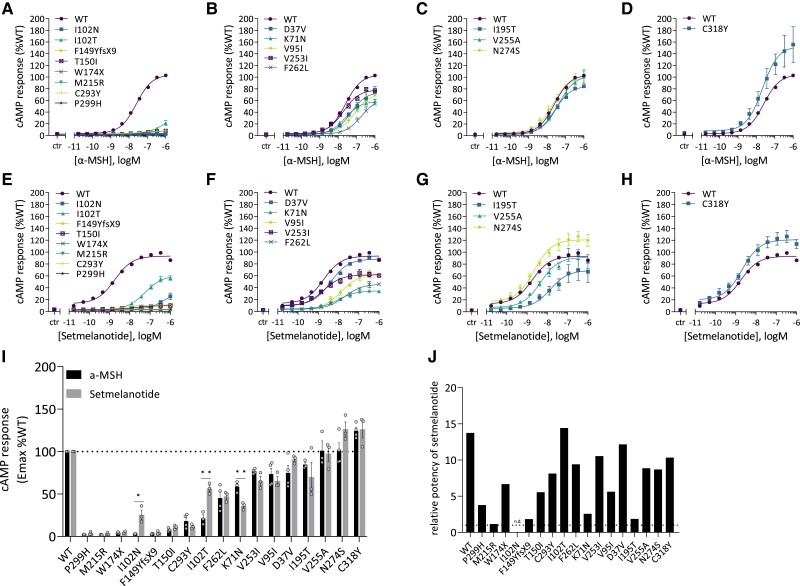
Ligand-induced cAMP response in HEK293 cells transfected with the MC4R variants. Dose-response curves for α-MSH (A-D) and setmelanotide (E-H) were generated. The cAMP response is expressed as Emax relative to WT MC4R (%WT). Emax (%WT) was compared between α-MSH and setmelanotide (I). The relative potency of setmelanotide is expressed as the EC_50_ ratio of setmelanotide and α-MSH. For cLoF MC4R variants the EC_50_ could be calculated; therefore the relative potency is expressed as n.d. (J). Values represent mean ± SEM from 3 to 4 independent experiments performed in duplicate. Statistical analysis was performed using unpaired two-tailed *t*-test; confidence level = 95%; ^*^P ≤ .05 and **P ≤ .001. Abbreviations: α-MSH, α-melanocyte stimulating hormone; cLoF, complete loss of function; EC_50,_ median effective concentration; Emax, maximum effect; HEK293, human embryonic kidney 293; MC4R, melanocortin-4 receptor; n.d., not determined; WT, wild type.

To determine the impact of MC4R variants on receptor signaling, we next performed independent experiments to assess the canonical Gα_s_-dependent cAMP response upon ligand stimulation [Fig. 3A–3D and Supplementary Table S5 (38)]. Eight of the 17 variants (I102N, I102T, F149YfsX9, T150I, W174X, M215R, C293Y, and P299H) showed an α-MSH-induced cAMP response that was less than 20% of WT (*P* < .001) and were therefore classified as cLoF variants (Fig. 3A). Five variants (D37V, K71N, V95I, V253I, and F262L) showed a partial cAMP responses to α-MSH stimulation with an Emax between 20% and 80% of WT (all *P* < .001) and were classified as pLoF variants (Fig. 3B). Variants I195T, V255A, and N274S showed normal α-MSH-induced cAMP responses, with a mean Emax similar to WT (all *P* > .05) (Fig. 3C). Lastly, C318Y displayed an increased cAMP response (>120% of WT, *P* < .01) and was classified as a GoF variant (Fig. 3D). Based on their α-MSH-induced cAMP response, we classified these variants as cLoF, pLoF, WT-like, or GoF variants.

We also investigated the effects of setmelanotide on the cAMP response of these variants [Fig. 3E–3H and Supplementary Table S5 (38)]. Comparing the %WT Emax in response to α-MSH and setmelanotide (Fig. 3I) showed that setmelanotide increased the %WT Emax of 3 variants (I102N, I102T, and N274S) compared to α-MSH. For I102T, the mean Emax increased significantly from 21.3% ± 4.3 to 56% ± 3.7 (*P* < .05), changing it from a cLoF to a pLoF variant. Upon stimulation with setmelanotide, N274S behaved as a GoF variant; however, the mean %WT Emax (126% ± 8.5 of WT) was not significantly different from that upon stimulation with α-MSH (101% ± 8.9) (*P* > .05). Interestingly, for variant K71N, stimulation with setmelanotide resulted in a significantly lower %WT Emax relative to WT compared to α-MSH (58% ± 3.7 vs 36% ± 2.4, respectively, *P* < .05).

Furthermore, setmelanotide had a greater potency than α-MSH for several of the variants [Supplementary Table S4 (38)]. Analyzing the EC_50_ ratio for setmelanotide relative to α-MSH for those 10 MC4R variants that displayed pLoF to GoF in cAMP signaling in response to either α-MSH or setmelanotide showed that setmelanotide has a potency 2- to 15-fold greater than α-MSH (Fig. 3J).

#### MC4R variants have different effects on ligand-induced recruitment of β-arrestin-2

**Figure 4.**
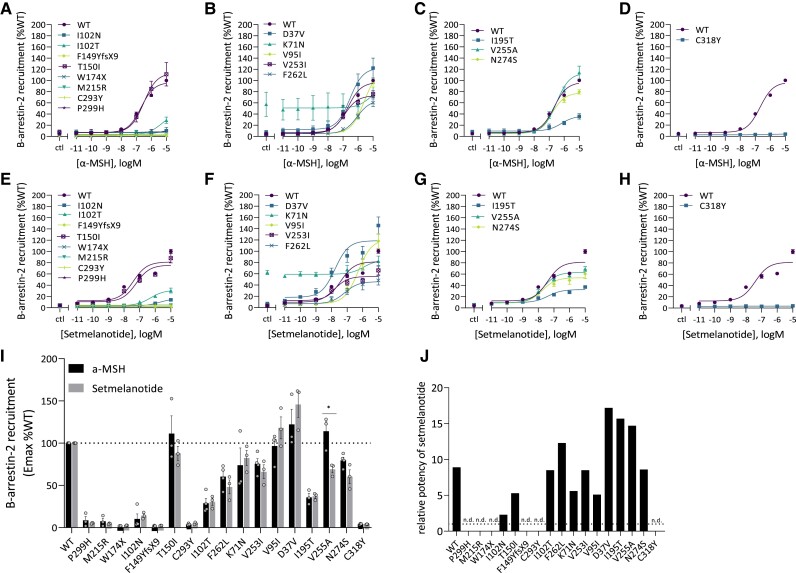
Ligand-induced β-arrestin-2 recruitment in HEK293 cells transfected with the MC4R variants. Dose-response curves for α-MSH (A-D) and setmelanotide (E-H) were generated. The β-arrestin-2 recruitment is expressed as Emax relative to WT MC4R (%WT). Emax (%WT) was compared between α-MSH and setmelanotide (I). The relative potency of setmelanotide is expressed as the EC_50_ ratio of setmelanotide and α-MSH. For cLoF MC4R variants the EC_50_ could be calculated; therefore the relative potency is expressed as n.d. (J). Values represent mean ± SEM from 3 to 4 independent experiments performed in duplicate. Statistical analysis was performed using unpaired two-tailed *t*-test; confidence level = 95%; ^*^P ≤ .05 and **P ≤ .001. Variants are ordered from cLoF, pLoF, WT-like, to GoF based on the α-MSH-induced cAMP response. Abbreviations: α-MSH, α-melanocyte stimulating hormone; cLoF, complete loss of function; EC_50,_ median effective concentration; Emax, maximum effect; GoF, gain of function; HEK293, human embryonic kidney 293; MC4R, melanocortin-4 receptor; n.d., not determined; pLoF, partial loss of function; WT, wild type.

To determine the impact of MC4R variants on ligand-induced recruitment of β-arrestin-2, we investigated the interaction between MC4R and β-arrestin-2 using the NanoBiT protein interaction assay [Fig. 4 and Supplementary Table S6 (38)]. First, we measured the recruitment of β-arrestin-2 to MC4R variants in response to α-MSH (Fig. 4A–4D). Most cLoF variants (I102N, F149YfsX9, T150I, W174X, M215R, C293Y, and P299H) had lost their ability to recruit β-arrestin-2 (*P* < .0001) in response to α-MSH (Fig. 4A). However, T150I showed WT-like levels of α-MSH-induced recruitment of β-arrestin-2 (*P* > .05). Most pLoF variants (K71N, V95I, V253I, and F262L) also showed a partial response to recruit β-arrestin-2 (all *P* > .05), although K71N displayed constitutive recruitment of β-arrestin-2 under basal conditions and was only partly responsive to ligand stimulation in the high micromolar range. D37V resulted in increased β-arrestin-2 recruitment (*P* > .05) (Fig. 4B). Of the variants with a normal cAMP response, V255A also displayed normal β-arrestin-2 recruitment (*P* > .05), while I195T and N274S yielded a partial loss of β-arrestin-2 recruitment (*P* < .001) (Fig. 4C). In particular for I195T, α-MSH-induced β-arrestin-2 recruitment was significantly reduced to 36% ± 4.8. Lastly, C318Y, a GoF variant, displayed a complete loss in β-arrestin-2 recruitment (*P* < .001) (Fig. 4D).

Second, analyzing the effect of setmelanotide on β-arrestin-2 recruitment by these MC4R variants, we found a pattern of effect of setmelanotide across the variants that was very similar to that of α-MSH [Fig. 4E–4I and Supplementary Table S6 (38)]. However, for 4 variants (D37V, I195T, F262L, and N274S), the potency of setmelanotide was increased 20- to 50-fold compared to 10-fold for the WT receptor, while for 4 other variants (K71N, V95I, I102N, and T150I) it was less potent in recruiting β-arrestin-2 (Fig. 4J). V255A resulted in significantly reduced efficacy but increased potency upon setmelanotide stimulation compared to α-MSH.

#### MC4R variants affect cell surface and total receptor expression

**Figure 5.**
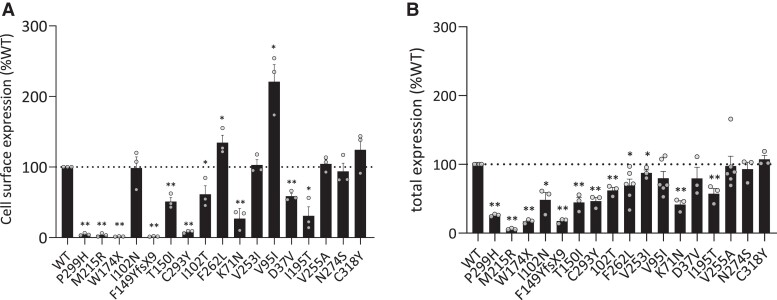
Cell surface expression (A) and total expression (B) of MC4R variants in HEK293 cells. The expression of the WT MC4R is set at 100% and variants are expressed as %WT. Values represent mean ± SEM from 3 to 4 independent experiments performed in quadruplicate. individual experiments are indicated by points in bar charts (two-tailed *t*-test; 95% confidence level; ^*^P ≤ .05 and **P ≤ .001). Variants are ordered from cLoF, pLoF, WT-like, to GoF based on the α-MSH-induced cAMP response. Abbreviations: α-MSH, α-melanocyte stimulating hormone; cLoF, complete loss of function; GoF, gain of function; HEK293, human embryonic kidney 293; MC4R, melanocortin-4 receptor; pLoF, partial loss of function; WT, wild type.

Next, we measured the cell surface expression and total levels of expression of the MC4R variants [Fig. 5A and Supplementary Table S7 (38), and 5B and Supplementary Table S8 (38), respectively]. Five cLoF variants (W174X, F149YfsX9, M215R, C293Y, and P299H) were undetectable at the cell surface (Fig. 5A), and also had low to undetectable total levels of expression (all *P* < .0001) (Fig. 5B). Two other cLoF variants (I102T and T150I) displayed reduced cell surface and total expression (*P* < .01). For the pLoF variants, D37V and K71N showed reduced cell surface expression (*P* < .001) combined with either normal (D37V) or reduced (K71N) total expression levels. I102N showed normal cell surface expression (*P* > .05) but a lower total expression level (*P* < .05). V95I, V253I, and F262L were expressed at the cell surface at significantly increased levels (*P* < .05), combined with either significantly reduced level (*P* < .05) (V253 and F262L) or normal (V95I) total expression levels. The WT-like variant I195T displayed significantly reduced cell surface expression (*P* < .05) and reduced total expression levels (*P* < .001). For the other WT-like variants, V255A and N274S, cell surface expression (*P* > .05) and total expression levels were not affected (*P* > .05). Also the GoF variant C318Y had a WT-like cell surface expression and total expression levels (*P* = .05).

#### Cell surface-expressed MC4R variants affect ERK signaling

**Figure 6.**
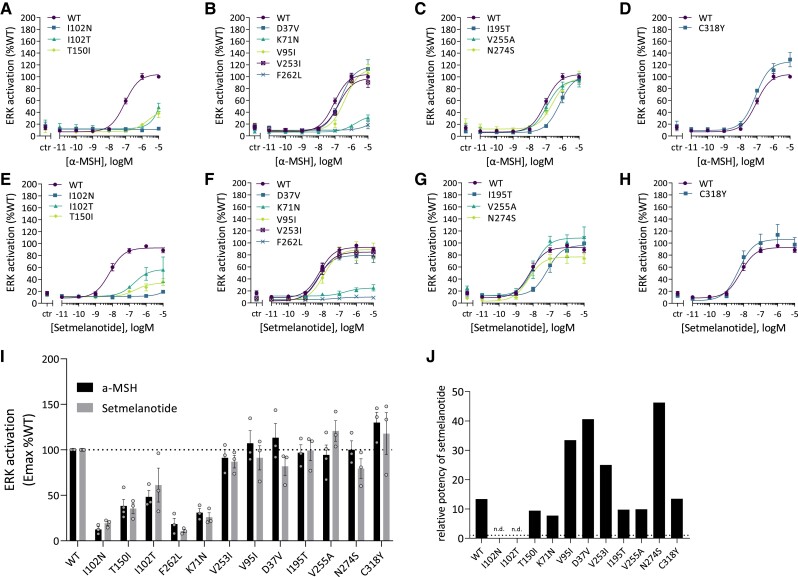
Ligand-induced ERK activation in HEK293 cells transfected with the MC4R variants. Dose-response curves for α-MSH (A-D) and setmelanotide (E-H) were generated. ERK activation is expressed as Emax relative to WT MC4R (%WT). Emax (%WT) was compared between α-MSH and setmelanotide (I). The relative potency of setmelanotide is expressed as the EC_50_ ratio of setmelanotide and α-MSH. For cLoF MC4R variants the EC_50_ could be calculated; therefore the relative potentcy is expressed as n.d. (J). Values represent mean ± SEM from 3 to 4 independent experiments performed in duplicate. Statistical analysis was performed using unpaired two-tailed *t*-test; confidence level = 95%; ^*^P ≤ .05 and **P ≤ .001. Variants are ordered from cLoF, pLoF, WT-like, to GoF based on the α-MSH-induced cAMP response. Abbreviations: α-MSH, α-melanocyte stimulating hormone; cLoF, complete loss of function; EC_50,_ median effective concentration; Emax, maximum effect; GoF, gain of function; HEK293, human embryonic kidney 293; MC4R, melanocortin-4 receptor; n.d., not determined; pLoF, partial loss of function; WT, wild type.

We next quantified the impact of MC4R variants on ligand-induced ERK activation. For this analysis, using the SRE-luciferase reporter assay, we selected those MC4R variants that displayed cell surface expression [Fig. 6A–6D and Supplementary Table S9 (38)]. Some cLoF or pLoF variants (K71N, 102N, I102T, T150I, and F262L) also showed reduced ERK activation in response to α-MSH (all *P* < .001). However, 3 pLoF variants (D37V, V95I, and V253I) displayed normal levels of ERK activation (*P* > .05). Correspondingly, 2 WT-like variants (V255A and N274S) also had WT-like levels of ERK activation relative to WT (all *P* > .05) in response to α-MSH. However, I195T, despite having a WT-like for cAMP response, showed a decrease in ERK activation (I195T *P* < .001). Finally, the GoF variant C318Y also displayed increased α-MSH-induced activation of ERK (*P* < .05).

Stimulation of MC4R variants with setmelanotide resulted in a very similar pattern of ERK activation [Fig. 6E–6H and Supplementary Table S9 (38)] compared to α-MSH. Lastly, we calculated the relative potency of α-MSH and setmelanotide for these MC4R variants to activate ERK (Fig. 6J). Setmelanotide was approximately 10-fold more potent than α-MSH. However, for variants D37V, V95I, V253I, and N274S, the potency of setmelanotide relative to α-MSH was further enhanced by 25- to 50-fold.

#### The majority of the patients with *MC4R* variants displayed a severe genetic obesity phenotype

There were 8 variants (I102N, I102T, T150I, W174X, F149YfsX9, M215R, C293Y, and P299H) that resulted in cLoF for the cAMP response from 6 pediatric patients and 2 adult patients (Table 1). Four out of these 6 pediatric patients had the highest BMI SDS in our study, ranging from +4.3 to +7.2 SD. The remaining 2 patients had a BMI SDS +3.1 and +3.5. The 2 adult patients had a BMI of 55.5 and 56.2 kg/m^2^, which were the highest in the adult group. The adult patient with the highest BMI (58.8 kg/m^2^) had the D37V variant. It should be noted that this patient also carries the pathogenic Y35X *MC4R* variant and that the D37V is commonly found on a haplotype with this nonsense mutation (47). Six out of 8 patients (2 adult and 4 pediatric) reported impaired satiety (Table 1). Most reported an age of obesity onset <5 years, while 1 patient reported an age of onset of 6.0 years. The age of obesity onset ranged between 0.3 and 6.0 years. All 8 patients reported obesity-related comorbidities [Supplementary Tables S3 and S4 (38)]. The 4 patients (2 pediatric and 2 adult patients) with pLoF variants for cAMP response showed a less profound hyperphagic phenotype, severity of obesity, and obesity-related comorbidities.

Besides the pediatric patient with a WT-like variant, I195T, showing no hyperphagia, the other 2 pediatric patients with WT-like variants exhibited a typical genetic obesity phenotype including an early age of onset of obesity and hyperphagia. All patients had similar obesity-related comorbidities compared to patients with cLoF variants. Lastly, the patient with a GoF variant for cAMP response, C318Y, displayed a BMI of 42.1 kg/m^2^, early-onset obesity, and evident hyperphagia. At 28 years of age, the patient had numerous obesity-related comorbidities, such as hyperinsulinemia, dyslipidemia, hypertension, polycystic ovary syndrome, type 2 diabetes, and elevated liver enzymes.

## Discussion

**Figure 7.**
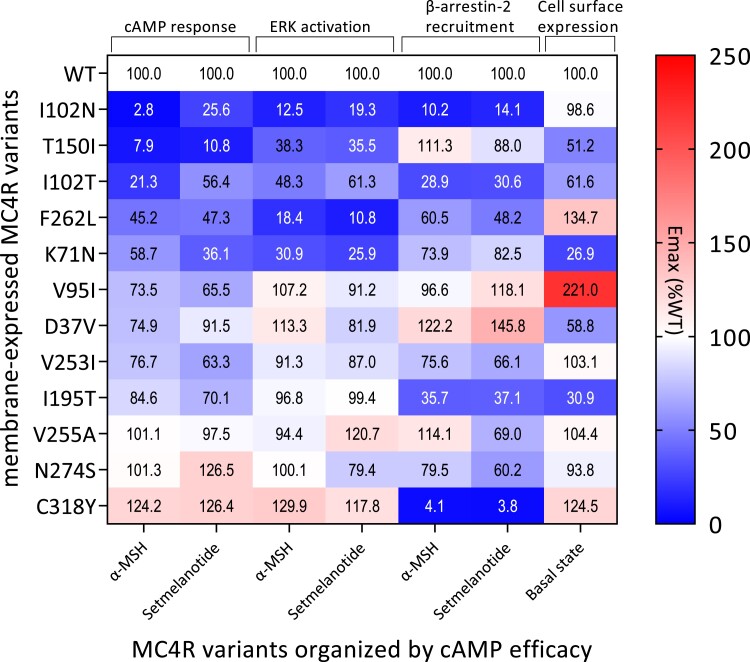
Summary of the effects of the MC4R variants on cell surface expression and α-MSH- or setmelanotide-induced cAMP response, ERK activation, and β-arrestin-2 recruitment. Variants are ordered from pLoF, WT-like, to GoF based on the α-MSH-induced cAMP response. Abbreviations: α-MSH, α-melanocyte stimulating hormone; GoF, gain of function; MC4R, melanocortin-4 receptor; pLoF, partial loss of function; WT, wild type.

In this study, we functionally characterized 17, predominantly heterozygous, *MC4R* variants identified in our patients with early-onset severe obesity. Six of the variants are novel, 3 have not been functionally characterized, and 8 variants have been reported previously but were incompletely characterized and reported as variants of unknown significance, likely pathogenic or pathogenic, according to ACMG guidelines (Table 2). We studied the response of these variant receptors to its endogenous ligand α-MSH but also to the synthetic agonist setmelanotide (Fig. 7). Setmelanotide has been approved by the Food and Drug Administration and European Medicines Agency for treatment of rare monogenic obesity caused by pathogenic variants in genes upstream of *MC4R* (48, 49). However, because setmelanotide has a greater potency for MC4R than α-MSH (35, 50), we analyzed whether setmelanotide could rescue signaling of the *MC4R* variants. Recent studies have shown that MC4R signaling can be mediated through several signaling pathways (30, 51). Pathogenic MC4R variants may therefore result in biased signaling (27), changing the response to its agonists and/or affecting only a subset of the downstream signaling pathways. Here we show that several of the studied MC4R variants differentially impact downstream signaling and the response to setmelanotide.

We identified 5 *MC4R* variants, 2 nonsense (F149YfsX9 and W174X) and 3 missense (P299H, M215R, and C293Y) variants, with a complete loss of cell surface expression. As expected, this also resulted in a complete loss of cAMP signaling and β-arrestin-2 recruitment in response to both α-MSH and setmelanotide. Thus these variants lead to a complete loss of function for all signaling pathways studied here and should be considered pathogenic and causative of obesity.

The other *MC4R* variants, however, showed a large heterogeneity in effects on studied endpoints (Fig. 7). Five variants (D37V, K71N, I102T, T150I, and I195T) showed reduced levels of cell surface expression combined with reduced total expression for most of them, likely resulting from impaired receptor trafficking (52, 53).

The variants K71N, I102T, and T150I also resulted in complete or partial loss of cAMP signaling with either normal or loss of β-arrestin-2 recruitment in response to α-MSH. However, K71N displayed significantly increased basal rates of β-arrestin-2 recruitment. This likely causes constitutive internalization and desensitization of the receptor (54), which is in line with the reduced cAMP signaling observed for this variant. T150I has been investigated previously, with similar outcomes to our study apart from showing a reduced β-arrestin-2 recruitment (27). A previous study identified I102T in normal weight subjects and functionally characterized it as a LoF variant based on reduced signaling and ligand binding (55), which our detailed functional analysis is in agreement with. I195T, despite having a lower cell surface expression, showed an α-MSH-induced cAMP response comparable to WT MC4R. This variant, however, was defective in β-arrestin-2 recruitment. We speculate that the receptor may have greater turnover at the cell surface, explaining the normal cAMP response in vitro. Another explanation could be that there are spare receptors in the in vitro assay system, as suggested by Tao (56). Spare receptors may still be able to respond to the ligand with maximum efficacy despite the reduction in surface expression caused by the I195T variant. However, the mechanism for this needs further investigation.

The remaining 7 variants had normal or increased cell surface expression with variable effects on cAMP response and β-arrestin-2 recruitment. Two of these variants (V255A and N274S) were comparable to WT MC4R for all pathways studied, which for N274S is in line with previous studies (30, 57). This raises the question whether these variants are pathogenic and thus associated with the obesity phenotype. Although clinically indistinguishable from patients carrying cLoF *MC4R* variants, there could still be alternative causes, including an unidentified variant in a different gene, polygenic obesity, or epigenetics. It should be noted that in a previous study V255A was reported to have a pLoF in cAMP response (75% Emax) and decreased binding (∼60%) to α-MSH compared to the WT receptor, although only the latter was significantly different from WT MC4R (58). Assay differences to measure cAMP possibly account for this difference.

It is also possible that other aspects of MC4R signaling should be studied to determine whether these are indeed pathogenic variants. It has been suggested that MC4R might signal through ERK (59-63). However, for V255A and N274S, α-MSH-induced ERK activation was comparable to activation by WT MC4R. In general, we observed that for all cell-surface expressed variants studied, the direction of effect of MC4R variants on ERK signaling was largely similar to that of the cAMP response and independent of the β-arrestin-2 recruitment findings. Variants with less than 80% α-MSH-induced cAMP response also displayed a strongly diminished ERK activation (<50%). However, variants with a partial reduction in cAMP response only mildly affected ERK signaling. This suggests that assessment of the cAMP response is more informative in determining the functional consequence of a variant.

Our results also suggest that the ERK and cAMP responses are likely both mediated through Gα_s_ and are not independent MC4R signaling events (30). We should be cautious to rule out biased effects on cAMP and ERK signaling as other variants may have different effects (58, 64). For instance, it has been reported that several MC4R variants, such as H76R, H158R, and L250Q, have normal ligand-induced ERK1/2 activity but constitutive activity for cAMP production (64, 65). Furthermore, ERK1/2 activity has also been shown to be induced by the Gα_q_/Ca^2+^ signaling pathway for other G-protein coupled receptors (66, 67). There is also evidence that MC4R can signal via Ca^2+^ ions (25, 68). Although Gα_s_ seems to be essential for ERK signaling, it could be that recruitment of other G proteins by MC4R could modulate this pathway. In addition, homo- and/or hetero-receptor dimerization (69) and interactions with MRAP2 (70) could be affected by the variants leading to altered biological function, but this remains to be explored.

Surprisingly, we identified 1 variant, C318Y, with a gain of function in cAMP response to both agonists. This variant is located in intracellular helix 8, shown to be important for proper cell surface localization and modulation of β-arrestin-2 binding (71, 72). The C318 residue has previously been shown to be lipid-modified, which may stabilize cell surface expression and the signaling capacity (73). Truncation of the receptor before C318 impaired cell surface expression, while truncation after C318 resulted in a normally expressed and functioning receptor. Truncation at C318 results in a receptor that can signal, although the receptor was rapidly internalized and cell surface expression could not easily be detected (73). Interestingly, the C318Y variant in our study caused a complete loss of β-arrestin-2 recruitment. We speculate that this results in retention of the receptor in the plasma membrane, explaining the increased signaling observed for this variant and underscoring the critical role of C318 in receptor function. However, the gain of function of this variant does not match with the obese phenotype of the patient as increased MC4R signaling is proposed to be associated with a lean phenotype (27).

Previously, MC4R variants, such as H76R, have been identified in patients with obesity that displayed constitutive activity, although some of these variants could not be further stimulated with α-MSH (65). In a study by Lotta et al, GoF *MC4R* variants were associated with significantly decreased BMI and risk of type 2 diabetes and coronary artery disease (27). The authors concluded that these beneficial associations are driven by increased β-arrestin-2 recruitment, rather than cAMP signaling.

At least 2 other obesogenic MC4R variants, R236C and T101N, have been described that, like C318Y, show bias toward the cAMP response, a suppressed ability to recruit β-arrestin-2, and increased cell surface expression (27, 74, 75). The effects of these variants reinforces the concept that β-arrestin-2 recruitment plays an important role in the regulation of body weight. However, the counterintuitive relationship these variants show between increased cAMP signaling and BMI demonstrates that further studies are needed to unravel the mechanism of their association with obesity. The use of patient-derived iPSC cells would aid to identify other pathogenic mechanisms, as it would allow the study the variant in the patient's own genetic background.

Despite the range of effects these variants had on the studied signaling pathways, we did not observe a clear correlation with the phenotype, although 6 out of 8 patients with cLoF variants for cAMP response had the highest BMIs. This could be explained by the fact that only patients with severe obesity and a medical or family history suggesting genetic obesity are referred to our clinic, and genetic screening is only performed when certain genetic obesity features are present. We observed that the majority of the patients displayed obesity-related comorbidities despite their young age at assessment. Furthermore, since we only had 1 patient per genotype, we are also unable to determine the penetrance of most of the variants. This is of interest, since recent studies indicate that other genetic factors contribute to the phenotype. For instance, a UK Biobank study showed that carriers of pathogenic *MC4R* variants with normal weight had a significantly lower polygenic risk score for BMI, compared to carriers with obesity (76). This illustrates that the polygenic background can modify the disease penetrance of monogenic variants.

Studies have shown that setmelanotide can be a potent weight loss therapy for patients with bi/allelic pathogenic *LEPR* and *POMC* variants (77). However, it is still unclear whether setmelanotide can lead to clinically relevant weight loss in patients carrying heterozygous pathogenic *MC4R* variants. Our study confirms that setmelanotide has a markedly greater potency, but similar efficacy, to α-MSH for WT MC4R cAMP signaling (35, 46), β-arrestin-2 recruitment, and ERK signaling. Thus, setmelanotide might potentiate the response of the unaffected allele and thereby be effective in heterozygous carriers. Indeed, heterozygous *Mc4r* knockout mice lose weight on setmelanotide treatment although they are less sensitive than WT mice (35). A study with a small group of patients harboring heterozygous *MC4R* variants showed that setmelanotide treatment resulted in weight loss (35). The precise mechanisms remains to be determined as it has also been suggested that agonists might restore the expression of mutant receptors at the cell surface (35). Whether setmelanotide elicits this effect on all or certain *MC4R* variants remains to be determined.

For most of the variants, we noticed that the cAMP Emax response to setmelanotide and α-MSH were similarly affected. However, for both I102N and I102T, setmelanotide improved the efficacy of the receptor to induce cAMP. In particular for I102T, stimulation with setmelanotide changed the cAMP response from a cLoF to a pLoF. Combined with the complete loss of β-arrestin-2 recruitment that these variants displayed for α-MSH and setmelanotide, this may suggest that setmelanotide may partially rescue signaling of these variants. Interestingly, the I102 variants are located close to the ligand binding pocket, particularly T101, which interacts directly with α-MSH and setmelanotide via a hydrogen bond (25, 78). Moreover, variants at T100 and V103 that are more responsive to ligand were recently shown to alter the conformation of setmelanotide allowing it to fit within the ligand binding pocket and/or to increase the binding affinity of setmelanotide (46). This also applies to the K71N and I195T variants. Setmelanotide did not alter the constitutive β-arrestin-2 recruitment of K71N; however, this variant significantly reduced the efficacy and potency of setmelanotide for cAMP signaling. I195T also displayed a reduced potency for setmelanotide-induced cAMP signaling. These results indicate that detailed analysis of distinct *MC4R* variants is needed to determine whether setmelanotide has beneficial effects beyond bi/allelic *POMC*, *PCSK1*, and *LEPR* loss of function variants. This could then be extended to clinical studies of patients with specific variants.

In conclusion, our study highlights the importance of studying the effect of variants on multiple aspects of MC4R signaling to fully understand the impact of a specific variant. In silico tools to predict the effects of these variants can be insufficient, as information on various signaling pathways is still incomplete. Indeed, our functional analyses show that several of the MC4R variants reported as likely pathogenic or of unknown significance according to the ACMG guidelines are partial or complete LoF variants (Table 2). We have categorized these variants based on the α-MSH induced cAMP response and also according to the molecular classification of MC4R mutations proposed by Tao (56), which indicates the functional consequence of the variants (Table 2). Knowledge about the impact of a distinct *MC4R* variant on MC4R signaling may aid in selecting the best personalized obesity treatment option. Although setmelanotide is less effective in patients with pathogenic *MC4R* variants, knowledge about the effect of a certain variant on the setmelanotide response may aid in identifying those patients that might benefit from this targeted pharmacological treatment.

## Data Availability

Restrictions apply to the availability of some or all data generated or analyzed during this study to preserve patient confidentiality. The corresponding author will on request detail the restrictions and any conditions under which access to some data may be provided.

## References

[1] Gavini CK , Cook TM, Rademacher DJ, Mansuy-Aubert V. Hypothalamic C2-domain protein involved in MC4R trafficking and control of energy balance. Metabolism. 2020;102:153990. 31666192 10.1016/j.metabol.2019.153990

[2] Kleinendorst L , Massink MPG, Cooiman MI, et al Genetic obesity: next-generation sequencing results of 1230 patients with obesity. J Med Genet. 2018;55(9):578‐586. 29970488 10.1136/jmedgenet-2018-105315

[3] Bray GA , Kim KK, Wilding JPH, World Obesity F. Obesity: a chronic relapsing progressive disease process. A position statement of the World Obesity Federation. Obes Rev. 2017;18(7):715‐723. 28489290 10.1111/obr.12551

[4] Farooqi IS , Keogh JM, Yeo GS, Lank EJ, Cheetham T, O'Rahilly S. Clinical spectrum of obesity and mutations in the melanocortin 4 receptor gene. N Engl J Med. 2003;348(12):1085‐1095. 12646665 10.1056/NEJMoa022050

[5] Polderman TJ , Benyamin B, de Leeuw CA, et al Meta-analysis of the heritability of human traits based on fifty years of twin studies. Nat Genet. 2015;47(7):702‐709. 25985137 10.1038/ng.3285

[6] Farooqi IS . EJE prize 2012: obesity: from genes to behaviour. Eur J Endocrinol. 2014;171(5):R191‐R195. 25127711 10.1530/EJE-14-0684

[7] Ignatieva EV , Afonnikov DA, Saik OV, Rogaev EI, Kolchanov NA. A compendium of human genes regulating feeding behavior and body weight, its functional characterization and identification of GWAS genes involved in brain-specific PPI network. BMC Genet. 2016;17(S3):158. 28105929 10.1186/s12863-016-0466-2 PMC5249002

[8] Yong Y , Cakir I, Lining Pan P, et al Endogenous cannabinoids are required for MC4R-mediated control of energy homeostasis. Proc Natl Acad Sci U S A. 2021;118(42):e2015990118. 10.1073/pnas.2015990118 PMC8545488 34654741

[9] Sweeney P , Chen C, Rajapakse I, Cone RD. Network dynamics of hypothalamic feeding neurons. Proc Natl Acad Sci U S A. 2021;118(14):e2011140118. 10.1073/pnas.2011140118 PMC8040641 33795520

[10] Tao YX . The melanocortin-4 receptor: physiology, pharmacology, and pathophysiology. Endocr Rev. 2010;31(4):506‐543. 20190196 10.1210/er.2009-0037 PMC3365848

[11] Doulla M , McIntyre AD, Hegele RA, Gallego PH. A novel MC4R mutation associated with childhood-onset obesity: a case report. Paediatr Child Health. 2014;19(10):515‐518. 25587224 10.1093/pch/19.10.515 PMC4276379

[12] Delhanty PJ , Bouw E, Huisman M, et al Functional characterization of a new human melanocortin-4 receptor homozygous mutation (N72K) that is associated with early-onset obesity. Mol Biol Rep. 2014;41(12):7967‐7972. 25163632 10.1007/s11033-014-3691-7

[13] Stutzmann F , Tan K, Vatin V, et al Prevalence of melanocortin-4 receptor deficiency in Europeans and their age-dependent penetrance in multigenerational pedigrees. Diabetes. 2008;57(9):2511‐2518. 18559663 10.2337/db08-0153 PMC2518504

[14] O'Rahilly S , Farooqi IS. Genetics of obesity. Philos Trans R Soc Lond B Biol Sci. 2006;361(1471):1095‐1105. 16815794 10.1098/rstb.2006.1850 PMC1642700

[15] Santoro N , Cirillo G, Xiang Z, et al Prevalence of pathogenetic MC4R mutations in Italian children with early onset obesity, tall stature and familial history of obesity. BMC Med Genet. 2009;10(1):25. 19284607 10.1186/1471-2350-10-25 PMC2664798

[16] Huszar D , Lynch CA, Fairchild-Huntress V, et al Targeted disruption of the melanocortin-4 receptor results in obesity in mice. Cell. 1997;88(1):131‐141. 9019399 10.1016/s0092-8674(00)81865-6

[17] Harrold JA , Williams G. Melanocortin-4 receptors, beta-MSH and leptin: key elements in the satiety pathway. Peptides. 2006;27(2):365‐371. 16290320 10.1016/j.peptides.2005.01.030

[18] Fani L , Bak S, Delhanty P, van Rossum EFC, van den Akker ELT. The melanocortin-4 receptor as target for obesity treatment: a systematic review of emerging pharmacological therapeutic options. Int J Obesity. 2014;38(2):163‐169. 10.1038/ijo.2013.80 23774329

[19] MacKenzie RG . Obesity-associated mutations in the human melanocortin-4 receptor gene. Peptides. 2006;27(2):395‐403. 16274851 10.1016/j.peptides.2005.03.064

[20] Lubrano-Berthelier C , Cavazos M, Dubern B, et al Molecular genetics of human obesity-associated MC4R mutations. Ann N Y Acad Sci. 2003;994(1):49‐57. 12851297 10.1111/j.1749-6632.2003.tb03161.x

[21] Hinney A , Volckmar AL, Knoll N. Melanocortin-4 receptor in energy homeostasis and obesity pathogenesis. Prog Mol Biol Transl Sci. 2013;114:147‐191. 23317785 10.1016/B978-0-12-386933-3.00005-4

[22] Drabkin M , Birk OS, Birk R. Heterozygous versus homozygous phenotype caused by the same MC4R mutation: novel mutation affecting a large consanguineous kindred. BMC Med Genet. 2018;19(1):135. 30068297 10.1186/s12881-018-0654-1 PMC6090656

[23] Wade KH , Lam BYH, Melvin A, et al Loss-of-function mutations in the melanocortin 4 receptor in a UK birth cohort. Nat Med. 2021;27(6):1088‐1096. 34045736 10.1038/s41591-021-01349-y PMC7611835

[24] Rene P , Lanfray D, Richard D, Bouvier M. Pharmacological chaperone action in humanized mouse models of MC4R-linked obesity. JCI Insight. 2021;6(4):e132778. 33434184 10.1172/jci.insight.132778 PMC7934941

[25] Heyder NA , Kleinau G, Speck D, et al Structures of active melanocortin-4 receptor-Gs-protein complexes with NDP-alpha-MSH and setmelanotide. Cell Res. 2021;31(11):1176‐1189. 34561620 10.1038/s41422-021-00569-8 PMC8563958

[26] Gillyard T , Fowler K, Williams SY, Cone RD. Obesity-associated mutant melanocortin-4 receptors with normal Galphas coupling frequently exhibit other discoverable pharmacological and biochemical defects. J Neuroendocrinol. 2019;31(10):e12795. 31529534 10.1111/jne.12795

[27] Lotta LA , Mokrosinski J, de Oliveira E M, et al Human gain-of-function MC4R variants show signaling bias and protect against obesity. Cell. 2019;177(3):597‐607 e599. 31002796 10.1016/j.cell.2019.03.044 PMC6476272

[28] Rajagopal S , Shenoy SK. GPCR desensitization: acute and prolonged phases. Cell Signal. 2018;41:9‐16. 28137506 10.1016/j.cellsig.2017.01.024 PMC5533627

[29] Fessikh M E , Belghiti H, Elkarhat Z, Guerinech H, Dakka N, El Baghdadi J. Identification of p.Met215Ile mutation of the MC4R gene in a Moroccan woman with obesity. Clin Case Rep. 2021;9(11):e05059. 34815872 10.1002/ccr3.5059 PMC8593808

[30] Brouwers B , de Oliveira EM, Marti-Solano M, et al Human MC4R variants affect endocytosis, trafficking and dimerization revealing multiple cellular mechanisms involved in weight regulation. Cell Rep. 2021;34(12):108862. 33761344 10.1016/j.celrep.2021.108862 PMC7994375

[31] Damm E , Buech TR, Gudermann T, Breit A. Melanocortin-induced PKA activation inhibits AMPK activity via ERK-1/2 and LKB-1 in hypothalamic GT1-7 cells. Mol Endocrinol. 2012;26(4):643‐654. 22361823 10.1210/me.2011-1218 PMC5417139

[32] Ramirez D , Saba J, Carniglia L, Durand D, Lasaga M, Caruso C. Melanocortin 4 receptor activates ERK-cFos pathway to increase brain-derived neurotrophic factor expression in rat astrocytes and hypothalamus. Mol Cell Endocrinol. 2015;411:28‐37. 25892444 10.1016/j.mce.2015.04.008

[33] Sutton GM , Duos B, Patterson LM, Berthoud HR. Melanocortinergic modulation of cholecystokinin-induced suppression of feeding through extracellular signal-regulated kinase signaling in rat solitary nucleus. Endocrinology. 2005;146(9):3739‐3747. 15961554 10.1210/en.2005-0562

[34] Kuhnen P , Krude H, Biebermann H. Melanocortin-4 receptor signalling: importance for weight regulation and obesity treatment. Trends Mol Med. 2019;25(2):136‐148. 30642682 10.1016/j.molmed.2018.12.002

[35] Collet TH , Dubern B, Mokrosinski J, et al Evaluation of a melanocortin-4 receptor (MC4R) agonist (setmelanotide) in MC4R deficiency. Mol Metab. 2017;6(10):1321‐1329. 29031731 10.1016/j.molmet.2017.06.015 PMC5641599

[36] Ayers KL , Glicksberg BS, Garfield AS, et al Melanocortin 4 receptor pathway dysfunction in obesity: patient stratification aimed at MC4R agonist treatment. J Clin Endocrinol Metab. 2018;103(7):2601‐2612. 29726959 10.1210/jc.2018-00258 PMC7263790

[37] Clement K , Biebermann H, Farooqi IS, et al MC4R agonism promotes durable weight loss in patients with leptin receptor deficiency. Nat Med. 2018;24(5):551‐555. 29736023 10.1038/s41591-018-0015-9

[38] Rodríguez Rondón AV , Welling MS, van den Akker ELT, et al Data from: MC4R variants modulate α-MSH and setmelanotide induced cellular signaling at multiple levels. *Figshare*. Date of Deposit December 5, 2023. 10.6084/m9.figshare.24712464 PMC11403317 38567654

[39] Kleinendorst L , Abawi O, van der Voorn B, et al Identifying underlying medical causes of pediatric obesity: results of a systematic diagnostic approach in a pediatric obesity center. PLoS One. 2020;15(5):e0232990. 32384097 10.1371/journal.pone.0232990 PMC7209105

[40] Richards S , Aziz N, Bale S, et al Standards and guidelines for the interpretation of sequence variants: a joint consensus recommendation of the American college of medical genetics and genomics and the association for molecular pathology. Genet Med. 2015;17(5):405‐424. 25741868 10.1038/gim.2015.30 PMC4544753

[41] Chima L , Mulrooney HM, Warren J, Madden AM. A systematic review and quantitative analysis of resting energy expenditure prediction equations in healthy overweight and obese children and adolescents. J Hum Nutr Diet. 2020;33(3):373‐385. 32073189 10.1111/jhn.12735

[42] Schofield WN . Predicting basal metabolic rate, new standards and review of previous work. Hum Nutr Clin Nutr. 1985;39(Suppl 1):5‐41. 4044297

[43] Roza AM , Shizgal HM. The Harris Benedict equation reevaluated: resting energy requirements and the body cell mass. Am J Clin Nutr. 1984;40(1):168‐182. 6741850 10.1093/ajcn/40.1.168

[44] Kruizenga HM , Hofsteenge GH, Weijs PJ. Predicting resting energy expenditure in underweight, normal weight, overweight, and obese adult hospital patients. Nutr Metab (Lond). 2016;13(1):85. 27904645 10.1186/s12986-016-0145-3 PMC5121980

[45] Lohman T . Assessment of body composition in children. Pediatr Exerc Sci. 1989;1(1):19‐30. 36696627 10.1123/pes.1.1.19

[46] Hammad MM , Mohammad A, Alam-Eldin N, et al Structural analysis of setmelanotide binding to MC4R variants in comparison to wild-type receptor. Life Sci. 2022;307:120857. 35931197 10.1016/j.lfs.2022.120857

[47] Hinney A , Schmidt A, Nottebom K, et al Several mutations in the melanocortin-4 receptor gene including a nonsense and a frameshift mutation associated with dominantly inherited obesity in humans. J Clin Endocr Metab. 1999;84(4):1483‐1486. 10199800 10.1210/jcem.84.4.5728

[48] EU/3/18/2101 . Orphan Designation for the Treatment of Leptin Receptor Deficiency. European Medicines Agency; 2018.

[49] FDA . FDA approves First Treatment for Weight Management for People with Certain Rare Genetic Conditions. U.S. Food & Drug Administration; 2020.

[50] Reininghaus N , Paisdzior S, Hopfner F, et al A setmelanotide-like effect at MC4R is achieved by MC4R dimer separation. Biomolecules. 2022;12(8):1119. 36009013 10.3390/biom12081119 PMC9405727

[51] Paisdzior S , Dimitriou IM, Schope PC, et al Differential signaling profiles of MC4R mutations with three different ligands. Int J Mol Sci. 2020;21(4):1224. 32059383 10.3390/ijms21041224 PMC7072973

[52] Dunham JH , Hall RA. Enhancement of the surface expression of G protein-coupled receptors. Trends Biotechnol. 2009;27(9):541‐545. 19679364 10.1016/j.tibtech.2009.06.005 PMC2731006

[53] Ulloa-Aguirre A , Zarinan T, Dias JA, Conn PM. Mutations in G protein-coupled receptors that impact receptor trafficking and reproductive function. Mol Cell Endocrinol. 2014;382(1):411‐423. 23806559 10.1016/j.mce.2013.06.024 PMC3844050

[54] Gray DL , Allen JA, Mente S, et al Impaired beta-arrestin recruitment and reduced desensitization by non-catechol agonists of the D1 dopamine receptor. Nat Commun. 2018;9(1):674. 29445200 10.1038/s41467-017-02776-7 PMC5813016

[55] Tao YX , Segaloff DL. Functional analyses of melanocortin-4 receptor mutations identified from patients with binge eating disorder and nonobese or obese subjects. J Clin Endocrinol Metab. 2005;90(10):5632‐5638. 16030156 10.1210/jc.2005-0519

[56] Tao YX . Molecular mechanisms of the neural melanocortin receptor dysfunction in severe early onset obesity. Mol Cell Endocrinol. 2005;239(1-2):1‐14. 15975705 10.1016/j.mce.2005.04.012

[57] Tao YX , Segaloff DL. Functional characterization of melanocortin-4 receptor mutations associated with childhood obesity. Endocrinology. 2003;144(10):4544‐4551. 12959994 10.1210/en.2003-0524

[58] Huang H , Tao YX. Pleiotropic functions of the transmembrane domain 6 of human melanocortin-4 receptor. J Mol Endocrinol. 2012;49(3):237‐248. 23014839 10.1530/JME-12-0161

[59] Chai B , Li JY, Zhang W, Newman E, Ammori J, Mulholland MW. Melanocortin-4 receptor-mediated inhibition of apoptosis in immortalized hypothalamic neurons via mitogen-activated protein kinase. Peptides. 2006;27(11):2846‐2857. 16806584 10.1016/j.peptides.2006.05.005

[60] Daniels D , Patten CS, Roth JD, Yee DK, Fluharty SJ. Melanocortin receptor signaling kinase in vitro and through mitogen-activated protein in rat hypothalamus. Brain Res. 2003;986(1-2):1‐11. 12965224 10.1016/s0006-8993(03)03162-7

[61] He S , Tao YX. Defect in MAPK signaling as a cause for monogenic obesity caused by inactivating mutations in the melanocortin-4 receptor gene. Int J Biol Sci. 2014;10(10):1128‐1137. 25332687 10.7150/ijbs.10359 PMC4202029

[62] Wei H , Ahn S, Shenoy SK, et al Independent beta-arrestin 2 and G protein-mediated pathways for angiotensin II activation of extracellular signal-regulated kinases 1 and 2. Proc Natl Acad Sci U S A. 2003;100(19):10782‐10787. 12949261 10.1073/pnas.1834556100 PMC196880

[63] Smith JS , Pack TF. Noncanonical interactions of G proteins and beta-arrestins: from competitors to companions. FEBS J. 2021;288(8):2550‐2561. 33539669 10.1111/febs.15749

[64] Mo XL , Tao YX. Activation of MAPK by inverse agonists in six naturally occurring constitutively active mutant human melanocortin-4 receptors. Biochim Biophys Acta. 2013;1832(12):1939‐1948. 23791567 10.1016/j.bbadis.2013.06.006

[65] Botha R , Kumar SS, Grimsey NL, Mountjoy KG. A unique melanocortin-4-receptor signaling profile for obesity-associated constitutively active variants. J Mol Endocrinol. 2023;71(1):e230008. 37040537 10.1530/JME-23-0008 PMC10304906

[66] Mousseaux D , Le Gallic L, Ryan J, et al Regulation of ERK1/2 activity by ghrelin-activated growth hormone secretagogue receptor 1A involves a PLC/PKCvarepsilon pathway. Br J Pharmacol. 2006;148(3):350‐365. 16582936 10.1038/sj.bjp.0706727 PMC1751558

[67] Leroy D , Missotten M, Waltzinger C, Martin T, Scheer A. G protein-coupled receptor-mediated ERK1/2 phosphorylation: towards a generic sensor of GPCR activation. J Recept Signal Transduct Res. 2007;27(1):83‐97. 17365511 10.1080/10799890601112244

[68] Kumar SS , Ward ML, Mountjoy KG. Quantitative high-throughput assay to measure MC4R-induced intracellular calcium. J Mol Endocrinol. 2021;66(4):285‐297. 33739935 10.1530/JME-20-0285 PMC8111326

[69] Kleinau G , Muller A, Biebermann H. Oligomerization of GPCRs involved in endocrine regulation. J Mol Endocrinol. 2016;57(1):R59‐R80. 27151573 10.1530/JME-16-0049

[70] Chan LF , Webb TR, Chung TT, et al MRAP and MRAP2 are bidirectional regulators of the melanocortin receptor family. Proc Natl Acad Sci U S A. 2009;106(15):6146‐6151. 19329486 10.1073/pnas.0809918106 PMC2661846

[71] Kirchberg K , Kim TY, Moller M, et al Conformational dynamics of helix 8 in the GPCR rhodopsin controls arrestin activation in the desensitization process. Proc Natl Acad Sci U S A. 2011;108(46):18690‐18695. 22039220 10.1073/pnas.1015461108 PMC3219140

[72] Seyedabadi M , Gharghabi M, Gurevich EV, Gurevich VV. Receptor-Arrestin interactions: the GPCR perspective. Biomolecules. 2021;11(2):218. 33557162 10.3390/biom11020218 PMC7913897

[73] Moore BS , Mirshahi T. Genetic variants help define the role of the MC4R C-terminus in signaling and cell surface stability. Sci Rep. 2018;8(1):10397. 29991773 10.1038/s41598-018-28758-3 PMC6039487

[74] Calton MA , Ersoy BA, Zhang S, et al Association of functionally significant Melanocortin-4 but not Melanocortin-3 receptor mutations with severe adult obesity in a large north American case-control study. Hum Mol Genet. 2009;18(6):1140‐1147. 19091795 10.1093/hmg/ddn431 PMC2649015

[75] Buchbinder S , Bartsch U, Muller M, Zorn M, Nawroth PP, Schilling T. A novel missense mutation T101N in the melanocortin-4 receptor gene associated with obesity. Genet Mol Res. 2011;10(2):1042‐1049. 21710454 10.4238/vol10-2gmr948

[76] Chami N , Preuss M, Walker RW, Moscati A, Loos RJF. The role of polygenic susceptibility to obesity among carriers of pathogenic mutations in MC4R in the UK biobank population. PLoS Med. 2020;17(7):e1003196. 32692746 10.1371/journal.pmed.1003196 PMC7373259

[77] Clement K , van den Akker E, Argente J, et al Efficacy and safety of setmelanotide, an MC4R agonist, in individuals with severe obesity due to LEPR or POMC deficiency: single-arm, open-label, multicentre, phase 3 trials. Lancet Diabetes Endocrinol. 2020;8(12):960‐970. 33137293 10.1016/S2213-8587(20)30364-8

[78] Zhang HB , Chen LN, Yang DH, et al Structural insights into ligand recognition and activation of the melanocortin-4 receptor. Cell Res. 2021;31(11):1163‐1175. 34433901 10.1038/s41422-021-00552-3 PMC8563965

